# Effects of Greenshell™ mussel intervention on biomarkers of cartilage metabolism, inflammatory markers and joint symptoms in overweight/obese postmenopausal women: A randomized, double-blind, and placebo-controlled trial

**DOI:** 10.3389/fmed.2022.1063336

**Published:** 2022-12-05

**Authors:** Maryam Abshirini, Jane Coad, Frances M. Wolber, Pamela von Hurst, Matthew R. Miller, Hong Sabrina Tian, Marlena C. Kruger

**Affiliations:** ^1^School of Health Sciences, College of Health, Massey University, Palmerston North, New Zealand; ^2^School of Food and Advanced Technology, College of Sciences, Massey University, Palmerston North, New Zealand; ^3^Centre for Metabolic Health Research, Massey University, Palmerston North, New Zealand; ^4^School of Sport, Exercise and Nutrition, College of Health, Massey University, Auckland, New Zealand; ^5^Cawthron Institute, Nelson, New Zealand; ^6^Sanford Ltd., Auckland, New Zealand

**Keywords:** osteoarthritis, biomarker, inflammation, greenshell mussel, joint pain

## Abstract

**Objective:**

To investigate the effect of whole greenshell mussel (GSM) powder on biomarkers of cartilage metabolism, inflammatory cytokines, and joint symptoms in postmenopausal women with overweight/obesity and joint discomfort.

**Design:**

Fifty-five postmenopausal women with overweight/obesity were randomly assigned to receive 3 g/day whole GSM powder or placebo for 12 weeks. Cartilage turnover biomarkers urinary C-telopeptide of type II collagen (CTX-II) and serum cartilage oligomeric matrix protein (COMP) were measured at baseline, week 6 and 12. Plasma cytokines were measured at baseline and week 12. Joint pain and knee-related problems were assessed at baseline and week 12 using a 100 mm Visual Analogue Scale (VAS) and the Knee injury and Osteoarthritis Outcome Score (KOOS) questionnaire, respectively.

**Results:**

Forty-nine participants completed the study (GSM *n* = 25, placebo *n* = 24). After 12 weeks, urinary CTX-II showed no significant change over time or between the groups (interaction effect *P* = 0.1). However, in women with symptomatic knees, a significant difference was noted between the group (treatment effect *P* = 0.04), as it was lower in the GSM group compared to placebo group at week 6 (*P* = 0.04) and week 12 (*P* = 0.03). Serum COMP and plasma cytokines were not affected. GSM supplementation showed greater reduction in the VAS pain score than placebo (−13.2 ± 20.3 vs. −2.9 ± 15.9; *P* = 0.04). No significant change in KOOS domains between the two groups was observed.

**Conclusion:**

Oral supplementation of whole GSM powder at 3 g/day may slow down the degradation of type II collagen in postmenopausal women with symptomatic knees. GSM treatment conferred clinical benefit on overall joint pain. No significant effect was noted for inflammatory cytokines, suggesting that GSM may act within the joint microenvironment rather than at the systemic level.

**Clinical trial registration:**

[www.australianclinicaltrials.gov.au/clinical-trialregistries], identifier [ACTRN12620000413921p].

## Introduction

Osteoarthritis (OA) is characterized by progressive degradation of articular cartilage and loss of joint function and is considered the most common type of joint disease and leading cause of disability among the elderly (1). OA prevalence is higher among women compared to men and its incidence rises following menopause (2). Women also tend to have a greater severity of knee OA (3). This drastic increase in OA incidence among postmenopausal women is linked to estrogen, which declines after menopause. The presence of both alpha and beta estrogen receptors (ERα and ERβ) in cartilage indicates that the chondrocytes may respond to estrogen and thereby reduction in estrogen would influence the metabolism of chondrocytes (4). Furthermore, menopause is associated with weight gain and increased body mass index (BMI) which is highly correlated with risk of knee and hip OA (5, 6).

A report on the association between obesity and OA incidence for non-weight bearing joints indicates the involvement of obesity-related metabolic factors such as adipokines and pro-inflammatory cytokines (7). Interestingly, the roles of mechanical loading and inflammation in development of radiographic knee OA were found to be more relevant in overweight and obese women than men (8). Excessive fat tissue induces production and release of the adipokines and pro-inflammatory cytokines resulting in low-grade systemic inflammation (9).

In addition, it is well-documented that inflammatory immune cells are recruited into the synovial joint and are involved in initiation of pathological changes in the synovial joint and initiation of obesity-associated OA (10). Tumor necrosis factor-α (TNF-α), interleukin-1 beta (IL-1β), and interleukin-6 (IL-6) are involved in cartilage degradation and bone resorption. These cytokines stimulate the expression of the cartilage-degrading enzymes, matrix metalloproteinases (MMPs), while inhibiting the formation of type II collagen and other cartilage matrix components (11).

Molecules derived from synovial joint tissue, particularly cartilage, have been used as biochemical markers of OA to detect the early change in metabolic and chemical properties of cartilage or predict disease progression and treatment monitoring (12). Type II collagen is the main component of cartilage and makes up 90–95% of the total collagen in cartilage (13). Proteolysis of type II collagen results in fragments of C-terminal telopeptides of type II collagen (CTX-II), which is measured as a biomarker of cartilage degradation. Cartilage oligomeric matrix protein (COMP) is a non-collagen structural protein involved in stabilization of extracellular matrix through interaction with collagen fibrils (14). Urine CTX-II and serum COMP are the most frequently studied biomarkers and have shown the best performance across all available biomarkers for OA. Both markers have been shown to be elevated in patients with OA and are correlated with radiographic severity of OA (15, 16). Despite extensive research and development of various markers, no single gold standard biomarker that is specific and sensitive to the damaged tissue and OA progression has been identified; therefore measuring a panel of biomarkers is necessary to provide an accurate picture on joint tissue metabolism (12).

An extract from New Zealand green-lipped mussel (*Perna canaliculus*) known as greenshell mussel™ (GSM) was found to be beneficial for joint health and symptom-relieving of OA in animal (17) and human clinical trials (18). The inhibitory effect of omega-3 polyunsaturated fatty acids eicosapentaenoic acid (EPA) and docosahexaenoic acid (DHA) present in GSM on cyclooxygenase-2 (COX-2) and the 5-lipoxygenase (5-LOX) cascade suppress synthesis of Prostaglandin E2 (PGE2) and reduce the inflammatory response (19). Furthermore, these fatty acids can resolve the existing inflammation through specialized pro-resolving mediators (SPMs) or derived compounds from EPA and DHA metabolized by LOXs. These novel anti-inflammatory molecules promote resolution of inflammation, tissue healing and relief of the chronic pain in rheumatic diseases (20, 21).

Recently, the cartilage protective activity of GSM has been demonstrated in a rat model of metabolic OA (22). The short-term pre-clinical study revealed that feeding with whole GSM powder decreased plasma levels of CTX-II, in rats fed a high-fat/high-sugar (HFHS) diet, indicating a preventive effect of GSM on cartilage degradation (22). Interestingly, in a matching long-term trial, rats were ovariectomized (OVX) to establish a model of post-menopausal OA combined with diet-induced obesity. Histopathological assessment of cartilage in knee joints demonstrated the Mankin score, a standard indicator of cartilage damage severity, was reduced in GSM-fed rats (23). Based on these observations, whole GSM powder has the potential to reduce type II collagen degradation and thereby attenuate the progression of OA in human subjects.

Previous studies have mainly focused on symptom-modifying effects of GSM extracts among OA patients and there is a lack of clinical studies using cartilage metabolism biomarkers to measure the GSM chondroprotective efficacy.

The clinical diagnosis of OA is usually made once disease is at late stage and most likely irreversible. Thus, this study targeted postmenopausal women with overweight/obesity with joint discomfort who are at risk or with early stage of OA, when an intervention is more likely to be beneficial. The current study a randomized, assessor and patient blinded, placebo-controlled trial aimed to investigate whether 12 weeks of supplementation with whole meat GSM powder supplementation affects the levels of cartilage metabolism biomarkers (primary outcome) and inflammatory cytokines along with joint pain and knee-related symptoms and function (secondary outcome) in overweight/obese postmenopausal women with joint discomfort. The placebo group was included for control. This study hypothesized that supplementation with GSM powder will decrease cartilage degradation biomarkers; urinary CTX-II and serum COMP and result in reduction of inflammatory cytokine levels, joint pain score and knee-related symptoms compared to placebo.

## Materials and methods

### Study participants

A total of 55 New Zealand women aged 55–75, ≥5 years post-menopause (based on the natural cessation of menstruation), with self-reported body mass index (BMI) between 25 and 35 kg/m^2^ (weight status was evaluated according to definition provided by Centre of Disease Control and Prevention: BMI 25 to <30 and 30–35 fell under overweight and obese, respectively) (24), and living in the Manawatū-Whanganui area were included. Participants reporting joint pain or discomfort within ≥3 months prior to study commencement without daily use of analgesic medicine were included. Subjects were excluded if they had a formal diagnosis of clinical OA, inflammatory arthritis or rheumatoid arthritis (RA), diabetes mellitus, or atherosclerosis, having chronic liver or renal disorder detected based on the screening blood test, having allergy to mussels or seafood, history of recent joint injury or trauma, smoking or having alcohol intake of more than two units per day, being on hormone replacement therapy for <6 months prior to the beginning of the trial, or taking anti-inflammatory drugs (glucocorticoids or NSAIDs) on a daily basis.

### Study design

A 12-week randomized; blinded, placebo-controlled study design was conducted. Women who met the initial inclusion criteria were screened by a routine non-fasted blood test for liver and kidney function, blood glucose (HbA1c), and lipid profile including triglycerides, total cholesterol, HDL-cholesterol, LDL-cholesterol at MedLab Central, Palmerston North, New Zealand. The purpose of routine blood tests was to screen the potential participants for liver and kidney disease and were repeated at the end of study to assess the safety of supplement. Prior to the baseline visit, participants who were regularly consuming oily fish (more than one meal per week) or taking fish oil or other joint health supplements were required to undergo a 4-week washout period. Then participants were randomly allocated into two groups, each consuming six capsules per day for 12 weeks: whole meat GSM powder (3 g/day) or placebo (sunflower seed protein). The subjects were instructed to consume the capsules with or after their meals.

The flash-dried whole meat GSM powder used in this study was comprised of 41.4% protein, 30.8% carbohydrate, 10.1% fat (EPA and DHA was 20.7 and 8% total fatty acids, respectively), 10.7% ash, and 7% moisture. The dose of 3 g/day was selected as it is achievable through diet (equivalent to 1–2 mussels) and this dose and duration were comparable to previous studies using whole GSM extracts in knee OA patients which resulted in pain improvement without any major adverse side effects (18). Flash dried whole meat GSM powder was produced by Sanford Ltd (PernaUltra™, Sanford, Blenheim, New Zealand) using standard manufacturing processes. Sunflower seed protein (BP Bulk powders, Braeside, Melbourne, Australia) was used as placebo as a neutral source of protein and was selected to be relatively similar to GSM powder in respect to macronutrient composition (66.6% carbohydrate, 24.3% protein, 3% fat, 3.4% moisture, and 2.7% ash) and to be as inert and non-bioactive as possible. Both GSM powder and placebo were encapsulated in hard-shell capsules by a commercial facility (Alaron, Nelson NZ) and stored under nitrogen in the dark at room temperature or lower until use. The GSM and placebo capsules were matched in the shape, size, and color of hard-shell encapsulant. Activated carbon sachets for absorbing moisture and odor were put in bottles to conceal any “fishy” odor. The nutritional composition and fatty acids profile of GSM powder and placebo used in the study is presented in Supplementary Table 1.

**FIGURE 1:**
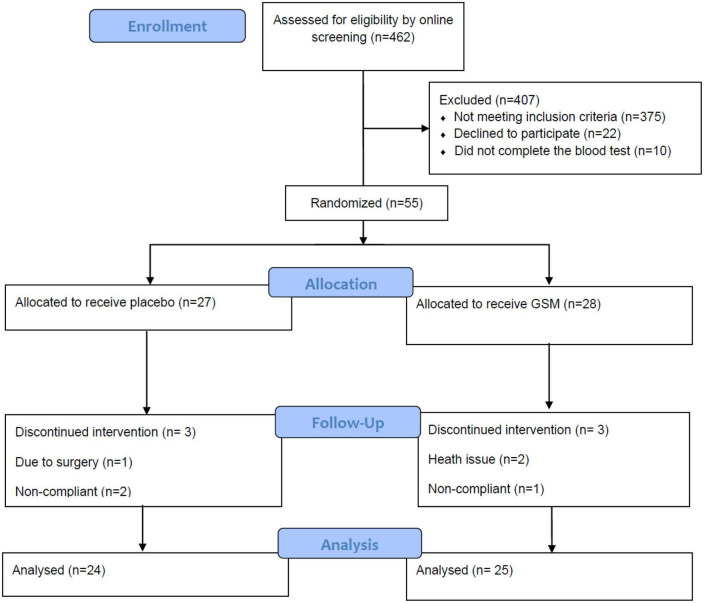
Schematic diagram of study design.

A randomization list was generated by Excel and maintained by project’s supervising investigator, who did not interact with the study subjects or conduct the primary data analysis. Randomization was stratified based on BMI (overweight: 25–29.9 kg/m^2^ and obese: 30–35 kg/m^2^) and age (55–64, 65–75 years) distribution. The primary researcher who was blinded to treatments code allocated participants to two supplements (A and B). Participants were blinded to treatment group until all analyses were completed. Data were collected during participants’ visit at baseline, follow-up (week 6) and end of the study (week 12) as shown in Figure 1.

Recruitment, screening, and data collection took place at the Human Nutrition Research Unit (HNRU) at Massey University, Palmerston North, New Zealand from August 2020 to September 2021.

### Demographic, anthropometric, and physical activity measurement

At baseline, participants completed a demographic questionnaire as well as anthropometric measurements including body weight and standing height measured using a beam balance to the nearest 0.2 kg and stadiometer to the nearest 0.1 cm, respectively. Body mass index (BMI) was calculated as weight (kg) divided by height squared (m^2^). Physical activity was assessed by the New Zealand Physical Activity Questionnaire – Short Form (NZPAQ-SF) (25). The NZPAQ has been validated by Boon et al. (26), and physical activities were computed by metabolic equivalent of task (METs)-min/week, which was calculated by the scoring protocol of International Physical Activity Questionnaire (IPAQ) for continuous score (27).

Metabolic equivalent of task values and formula for calculation of MET-minutes were assessed and used as below:

• Walking MET-minutes/week at work = 3.3 × walking minutes × walking days at work.
• Moderate MET-minutes/week at work = 4.0 × moderate-intensity activity minutes × moderate intensity days at work.
• Vigorous MET-minutes/week at work = 8.0 × vigorous-intensity activity minutes × vigorous.
• Total Work MET-minutes/week = sum of Walking + Moderate + Vigorous MET-minutes/week scores at work.

### Dietary intake assessment

Each participant’s daily nutrient intake from the diet was measured using a 3-day food record including 2 weekdays and 1 weekend day at the midpoint of the trial. The 3-day food record has been recommended and considered as the “gold standard” for dietary assessment. Instructions on how to accurately complete the food record was provided (28). The brand name of food products, recipes, and food preparation were recorded. Each participant’s nutrient intake was calculated using Foodworks 9 Professional, Xyris Software.

### Biochemical analyses

Second void morning urine specimens were collected after overnight fasting at baseline, weeks 6 and 12 for assessment of CTX-II. Overnight fasting blood samples were collected by a certified phlebotomist at baseline, weeks 6 and 12 to measure COMP. Blood samples were collected into serum and EDTA-anticoagulated plasma vacutainer tubes. The blood sample tubes for serum collection, were incubated for 1 h at room temperature, followed by centrifugation at 2,264 g for 10 min at 4°C (Gyrozen 1248R Multi-Purpose High-Speed, Korea) to isolate the serum. The EDTA tubes were centrifuged immediately after blood drawing in the same manner to collect the plasma. Serum, plasma and urine samples were aliquoted and stored at −80°C until use.

Measurement of serum biomarkers was performed using commercially available enzyme linked immunoassay (ELISA) kits. Assays for serum cartilage oligomeric matrix protein (COMP) were performed with BioVendor Research and Diagnostic Products (Karasek, Czechia). The detection limit was 0.4 ng/ml. The intra-assay precision co-efficient of variation (CV) was 4.0–8.0% and the inter-assay precision CV was 3.1–6.6%.

Urinary CTX-II concentrations were determined using an enzyme immunoassay (EIA) kit (Urine CartiLaps^®^ EIA; Immunodiagnostic systems, Herlev, Denmark). Urinary creatinine (Cr) was measured by colorimetric method (RX Daytona+; Randox Laboratories Ltd.). Concentrations of urinary CTX-II were corrected by urinary Cr using the following formula: corrected CTX-II value (ng/mmol Cr) = 1,000 × urine CartiLaps (μg/L)/creatinine (mmol/L). The detection limit was 0.2 ng/ml. The intra-assay precision CV were 5.2, 4.6, and 7.8% for high, medium and low ranges of measurement. The inter-assay precision CV were 6.9, 10.8, and 12.2% for high, medium and low ranges of measurement. Serum COMP and urinary CTX-II were assessed in duplicate.

Plasma C-terminal telopeptide of type I collagen (CTX-1) and parathyroid hormone (PTH) at baseline were analyzed by electrochemiluminescence immunoassays using the Roche COBAS^®^ e411 system (Roche Diagnostics, Indianapolis, IN, USA). Cytokine assays were performed using BioLegend^®^ LEGENDplex Multi-Analyte Flow Assay following the kit instructions and measured using a Beckman Coulter Gallios flow cytometer. Levels of cytokines including TNF-α, IL-1β, IL-6, IL-4, IL-10, IL-15, and IL-18 were quantified in plasma at baseline and end of the study. Baseline level of plasma 25(OH) vitamin D were analyzed using isotope-dilution liquid chromatography-tandem mass spectrometry (ID-LC-MSMS) by Canterbury Health, Christchurch, New Zealand. Serum 25(OH)D ≥ 50 nmol/L at the end of winter, and 10–20 nmol/L higher at the end of summer to allow for seasonal variation, has been considered optimal for musculoskeletal health for people residing in Australia and New Zealand (29). Vitamin D insufficiency or deficiency in this study was considered as plasma 25(OH)D < 50 nmol/L.

### Self-assessment of pain visual analogue scale and knee injury and osteoarthritis outcome score

Secondary outcome measures including pain visual analogue scale (VAS) and knee injury and osteoarthritis outcome score (KOOS) were recorded at baseline and week 12. The pain levels were reported by participants using a 100 mm linear measure of pain status scored from 0 to 100 mm where 0 was defined as having no pain and 100 the worst pain ever experienced within the past week. Pain rated at ≥30 was regarded as having a moderate to high level of pain. This cut-off was selected based on the required entry criteria of a previous clinical trial (30). Participants with more than one joint site with pain completed the VAS for overall joint pain.

Knee Injury and Osteoarthritis Outcome Score is commonly utilized in research and clinical practice to measure short- and long-term consequences of knee problems (31). The previous week is the period included when answering the questions about the knee problem. It consists of 42 items which cover five domains: knee pain (Pain), other symptoms (Symptoms), activities of daily living (ADL), function in sport and recreation (Sport/Rec) and knee related quality of life (QOL). All items are scored on a 5-point Likert scale (0–4), and each domain is scored separately as the sum of all corresponding items. A total score has not been validated and is not recommended. Scores are then converted to a 0–100 scale (percentage of total possible score obtained), where 0 represents extreme knee problems and 100 represents no knee problems (32). This questionnaire was completed by those who reported knee pain and established cut-off score of ≤86 for any of the domains is used to classify individuals with symptomatic knees (33). The validity of KOOS has previously been demonstrated by construct and content and good to excellent test-retest reliability (34, 35).

### Compliance assessment

To assess subjects’ compliance, diaries were provided to participants at baseline to record their daily intake of study supplement and analgesic medications. Participants were allowed to continue taking paracetamol or any supplements that did not contain omega-3 fatty acids or chondroprotective bioactive compounds. Compliance assessment was performed using cumulative capsule counts at the completion of the study, and adherence was measured as a percentage: [(number of capsules provided minus number of unused capsules)/number of capsules provided] × 100. Adherence below 80% was considered a protocol violation.

Moreover, at the baseline and end of the study, the plasma and red blood cell membrane *n*-3 PUFA, EPA, DHA, and total *n*-3 L-C PUFA (including alpha-linolenic acid (ALA, 18:3 *n*-3), stearidonic acid (SDA, 18:4 *n*-3), eicosatetraenoic acid (ETA 20:4 *n*-3), EPA, docosapentaenoic acid (DPA,22:5 *n*-3) and DHA) were measured to assess the adherence to study protocol. The plasma and red blood cell *n*-3 PUFA were analyzed by gas chromatography (GC, Agilent Technologies Australia, VIC, Australia). Fatty acids were identified to an external commercial fatty acid standard. The analysis were done at the Cawthron Institute, Nelson, New Zealand and methodologies are published elsewhere (36).

### Safety assessment

Any adverse side effect was recorded by participants in their diaries. Participants documented the events by rating the severity (mild, moderate, and severe) and medications required to treat the events. Moreover, routine laboratory blood test including liver and kidney function tests, blood glucose (HbA1c) and lipid profile (triglyceride, total cholesterol, HDL-cholesterol, and LDL-cholesterol) were assessed from non-fasted venous blood samples at baseline and end of trial at MedLab Central Palmerston North, New Zealand.

### Statistical methods

The sample size was based on urine CTX-II/creatinine and serum COMP as the primary outcomes of the study. Sample size was calculated to detect 20% difference between the groups using the standard deviation from an unpublished report. For urine CTX-II/creatinine a sample size of 24 was required to detect a 20% relative difference from baseline with 80% power. For serum COMP a sample size of 17 was required to detect a 20% difference between groups with a power of 95%. The sample size of 48 (*n* = 24 per group) was required as a manageable sample size. Finally, a total sample size of 55 was needed to allow for at ∼10% potential dropout rate (*n* = 27–28 per group).

Statistical analysis was performed using IBM SPSS version 26.0 (Armonk, NY, USA). Analysis was conducted on the dataset from participants who completed assessment at both timepoints (baseline and endpoint). Variables were checked for normality using the Kolmogorov-Smirnov, Shapiro-Wilk tests and data that were not normally distributed were log-transformed. The data were reported as mean ± standard deviation (SD) for normally distributed data, and as median (25th, 75th percentiles) for non-normally distributed data, and as frequencies for categorical data. The baseline characteristics of subjects between two treatment groups were compared using Student’s *t*-test for parametric, and the Mann-Whitney *U* test for nonparametric data. Regarding the categorical variables, the distribution of participants was analyzed using the Chi-square tests or Fisher’s exact test where more than 20% of data cells had expected count below 5.

Missing data points were imputed with mean values of each group (the mean value of each group was assigned to those with missing data) to include all the data in the analysis. Outcome analyses were conducted on data with and without imputed missing values. Two-way repeated measures ANOVA was used to examine differences within each group over time (pre- vs. post-intervention) and between the groups (GSM vs. placebo). In case of significant effect, analysis was followed by post hoc comparison using the Tukey test. For analysis of cartilage markers, the data were analyzed on both mean value and value relative to baseline in order to reduce the variability and achieve the normal distribution. In order to control the effect of the main potential confounding factors, age and BMI on outcome measures, particularly cartilage markers, the treatment groups were stratified for these factors (37).

The interactions between treatments and time indicate differences in efficacy. For VAS pain and KOOS score analysis, covariates including baseline level of VAS pain or KOOS domain score, compliance, paracetamol use, and season of enrolment were adjusted in models. The relationships between cartilage degradation markers, plasma 25(OH)D, VAS, and KOOS domain score were assessed using Pearson correlation. Statistical significance was considered by two-sided *P* < 0.05.

## Results

### Baseline characteristics of participants

The flow diagram of study is presented in Figure 1. Initially 462 women filled a pre-screening online questionnaire, and the majority of them were excluded due to distance from the location of research or being diagnosed with health condition mentioned in the exclusion criteria. Finally, 66 women passed the online screening and were phone interviewed and invited for a blood test screen. From this group, 55 completed the blood test and were eligible for trial entry. Of the 55 enrolled participants, six subjects dropped out from the trial. Finally, a total of 49 participants (GSM, *n* = 25 and placebo, *n* = 24) completed the study. The baseline characteristics of the participants who completed the study are shown in Table 1. It is important to note that due to COVID-19 restrictions, one subject from the GSM group missed a follow-up visit. For blood markers analysis, blood samples were not available from two participants at week 6 from GSM group (one due to missing the visit due to COVID-19 lockdown and one due to phlebotomy issues). Urine samples were provided by all participants from both groups at all time points, except for one participant from the GSM group due to missing the visit at week 6.

**TABLE 1:** General characteristics of participants who completed the study.

General characteristics	Overall population (*n* = 49)	Placebo (*n* = 24)	GSM (*n* = 25)	*P*-value
Age (years), mean ± SD	63.5 ± 5.4	62.9 ± 5.4	64.2 ± 5.1	0.3
Height (cm), mean ± SD	164.8+6.7	164.7 ± 6.4	164.8 ± 7.1	0.8
Weight (kg), median (25th, 75th percentiles)	75.7 (68.3, 86.2)	73.8 (68.2, 88.6)	77.2 (68.5, 86.2)	0.9
BMI categories [*n* (%)]				0.6
Overweight	33 (67.4)	17 (70.8)	16 (64)	
Obese	16 (32.6)	7 (29.2)	9 (36)	
Physical activity (MET-minutes/week), median (25th, 75th percentiles)	764 (307.5, 1794)	751 (318, 2373.7)	764 (287, 1483)	0.1
Ethnicity [*n* (%)]				0.1
NZ European	44 (89.1)	20 (83.3)	24 (96)	
Māori/Other	5 (10.2)	4 (16.7)	1 (4)	
Season of enrolment [*n* (%)]				
Spring	7 (14.3)	6 (25)	1 (4)	
Summer	17 (34.7)	6 (25)	11 (44)	0.1
Autumn	22 (44.9)	10 (41.7)	12 (48)	
Winter	3 (6.1)	2 (8.3)	1 (4)	
Whole body *T*-score ≤2.5 [*n* (%)]	12 (24.5)	6 (25)	6 (24)	0.9
VAS pain score ≥ 30 [*n* (%)]	21 (42.9)	7 (29.2)	14 (56)	**0.05**
KOOS domain score ≤ 86 [*n* (%)]	39 (79.6)	18 (75)	21 (84)	0.4
Paracetamol use [*n* (%)]				0.2
Yes	14 (28.5)	5 (20.8)	9 (36)	
No	35 (71.4)	19 (79.2)	16 (64)	
Biochemical markers				
Plasma CTX-I (μg/L), mean ± SD	0.44 ± 0.14	0.46 ± 0.13	0.43 ± 0.16	0.4
Plasma 25(OH)D (nmol/L), median (25th, 75th percentiles)	73 (56, 83)	69.5 (46, 79.7)	78 (64, 87)	**0.007**
Plasma PTH (picomol/L), median (25th, 75th percentiles)	4.3 (3.5, 5.3)	4.7 (4.2, 6.4)	3.8 (3.5, 4.8)	**0.007**

BMI, body mass index (kg/m^2^); MET, metabolic equivalent of task; VAS, visual analogue scale; KOOS, knee injury and osteoarthritis outcome score; CTX-I, C-terminal telopeptides of type I collagen. Values are presented as mean ± standard deviation or median (25th and 75th percentile) for normally distributed and non-normally distributed variables, and *n* (%) for categorical variables for which the percentage within each treatment group is reported. Significance level (*P* < 0.05) is indicated in bold.

The two groups were similar at baseline with respect to most demographic characteristics. For the overall study population, the mean age was 63.5 ± 5.4 years; 67.4% of women were overweight (BMI between 25 and 29.9 kg/m^2^) and 32.6% were obese (BMI ≥ 30 kg/m^2^).

The median (25th, 75th percentile) of physical activity level was 751 (318, 2373.7) MET-minutes/week and 764 (287, 1483) MET-minutes/week in placebo and GSM group, respectively, with no significant difference between the groups.

The majority of participants (89.1%) were of European-New Zealand ethnicity. Paracetamol use during the study was reported by 14 (28.5%) of the participants and did not differ between groups.

Some significant differences were observed. Out of 49 women, 21 (42.9%) were characterized as having moderate to high levels of joint pain (VAS pain score ≥ 30) and this was significantly different between the groups with a higher proportion in the GSM group (56 vs. 29.2%, *P* = 0.05). In term of knee related problems, 39 (79.6%) women had knee symptoms (KOOS domain score ≤ 86). With respect to joint pain location, 18 (36.7%) had only knee pain, 21 (42.8%) had pain at knee and hip or other joints, and 10 (20.4%) reported pain at hand and/or back or shoulder.

The baseline level of plasma CTX-I was comparable between the two groups, while the baseline level of 25(OH)D and PTH were significantly different between the groups, as vitamin D level was higher and PTH was lower in the GSM group compared to the placebo group (*P* = 0.007). However, the percentage of participants with vitamin D insufficiency or deficiency [25(OH)D below 50 nmol/L] was not significantly different between the groups with 2 (8%) in the GSM group and 6 (25%) in the placebo having vitamin D levels below the normal range (*P* = 0.1).

Daily energy and nutrient intake of participants are presented in Supplementary Table 2. The daily nutrient intake of participants showed no differences between the groups. The average consumption of fish and seafood among the participants prior to enrolment to the study was less than once a week (60%), once a week (35%) and more than once a week (5%), and none were a regular mussel eater.

### Evaluation of treatment on cartilage degradation markers

**FIGURE 2:**
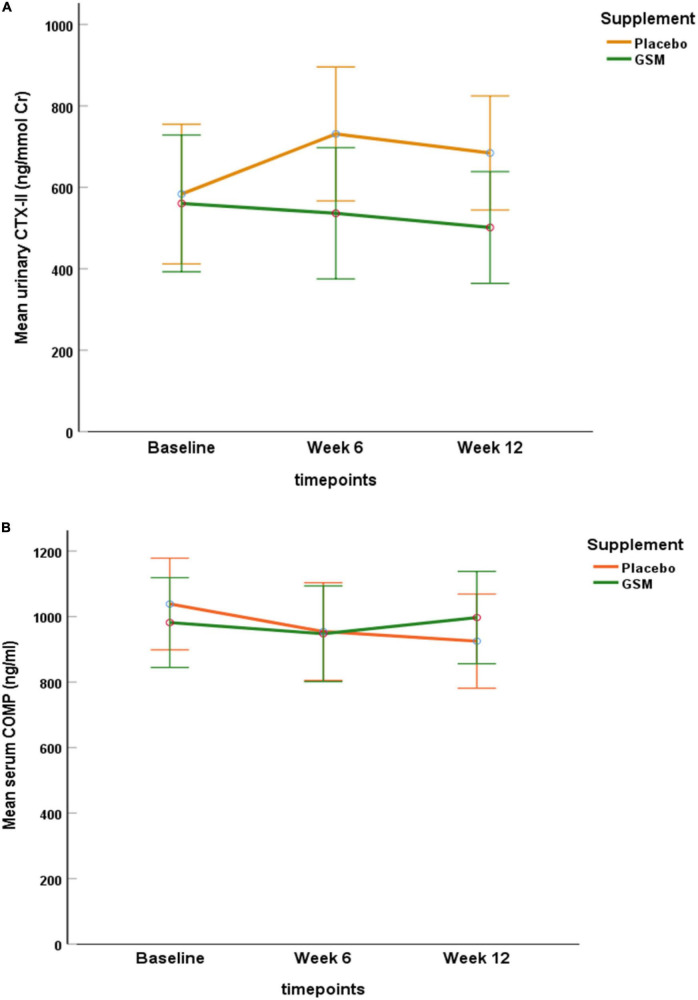
Pattern of change in urinary C-telopeptide of type II collagen (CTX-II) level **(A)**, and serum level of cartilage oligomeric matrix protein (COMP) **(B)** over the study period (baseline, follow-up, and endpoint) within each of the treatment groups. No significant neither over time nor between the groups for urine CTX-II and serum COMP (interaction effect *P* = 0.3, *P* = 0.1, respectively). Data are expressed as the mean ± standard error.

At baseline, the mean urinary CTX-II level was 560.4 ± 428 ng/mmol Cr in GSM group and 583.3 ± 411 ng/mmol Cr in placebo group and there was no significant difference between the groups (*P* = 0.8). As demonstrated in Figure 2A, the urinary CTX-II level slightly decreased from the baseline during intervention in the GSM group, while it notably increased from baseline and peaked at week 6 and then slightly reduced at week 12 in placebo group, however, the overall change was not significant between the groups (interaction effect *P* = 0.3).

The baseline level of serum COMP was 972.3 ± 272 and 1,040.4 ± 402 ng/ml in the GSM and placebo groups, respectively. As shown in Figure 2B, serum COMP trended to slightly decrease in placebo and increase in GSM group. Overall, it remained stable and did not change meaningfully over the study period or between group (interaction effect *P* = 0.1).

**FIGURE 3:**
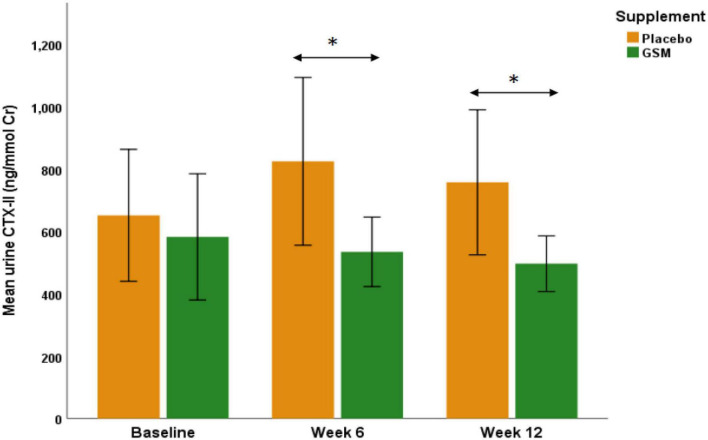
The urinary C-telopeptide of type II collagen (CTX-II) levels in those subjects with knee injury and osteoarthritis outcome score (KOOS) domain score of 86 or below in GSM group (*n* = 21) and placebo (*n* = 18). There was a significant difference between groups as urinary CTX-II was significantly lower in GSM compared to placebo at week 6 (*P* = 0.04) and week 12 (*P* = 0.03). The values are expressed at mean ± standard error and compared by Student’s *t*-test at each time point. *Indicates the significance (*P* < 0.05).

To further evaluate the effect of GSM supplementation on urinary CTX-II level, subjects with a KOOS domain score 86 or below were included in a further analysis (GSM, *n* = 21 and placebo, *n* = 18). The baseline characteristics of these subjects and level of cartilage degradation biomarkers were not statistically significant between the treatment groups (data not shown). As shown in Figure 3, in subjects with symptomatic knees, the urinary CTX-II showed similar pattern of change as overall population. The result of analysis on participants with KOOS below 86 showed urine CTX-II level were significantly different among the treatment groups during the intervention (treatment effect *P* = 0.04) with significantly lower levels in the GSM group compared to placebo at week 6 (534.6 ± 255.4 vs. 824.7 ± 570.4 ng/mmol Cr, *P* = 0.04) and end of the study (496.6 ± 204.2 vs. 757.4 ± 493.2 ng/mmol Cr, *P* = 0.03). However, there was no significant change over time within groups (time effect *P* = 0.9) and between groups (interaction effect *P* = 0.3).

The analysis for urine CTX-II and serum COMP was conducted on the data corrected for baseline as shown in Supplementary Figure 1. There was a significant change overtime (time effect *P* = 0.03), although the overall change was not significant between the groups (interaction effect *P* = 0.1; Supplementary Figure 1A). The result for serum COMP corrected for the baseline was similar to uncorrected as no significant effect was noted (Supplementary Figure 1B).

The result of analysis for data without imputing missing values were similar to the imputed data.

### Evaluation of treatment on visual analogue scale pain and knee injury and osteoarthritis outcome score domains

The baseline VAS pain score in the GSM group was 21.6 ± 15.9 and in the placebo group was 29.4 ± 21 and there was no significant difference between the two groups (*P* = 0.07).

**FIGURE 4:**
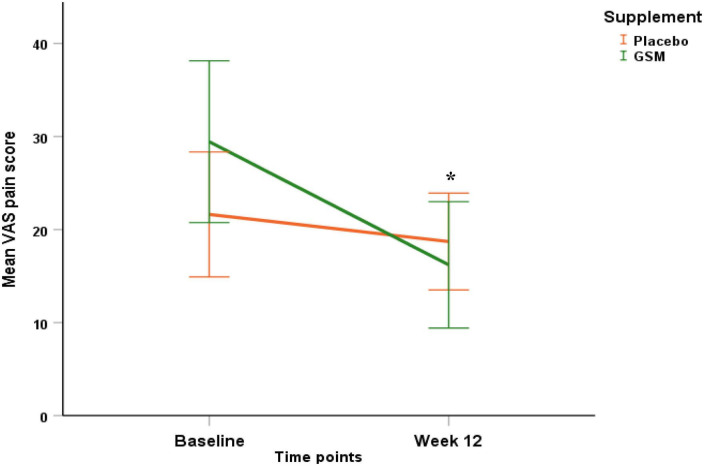
Pattern of change in visual analogue scale (VAS) pain score over the study period (baseline and endpoint) within each of the treatment groups. Significant effect of time (*P* = 0.002), and greater reduction in VAS pain score in GSM supplement compared to placebo (interaction effect *P* = 0.03 unadjusted and *P* = 0.04 adjusted for baseline level, compliance, use of paracetamol and season of enrolment). Placebo = orange, GSM = green. Values are expressed as mean (95% confidence interval). *Indicates the significance (*P* < 0.05).

**FIGURE 5:**
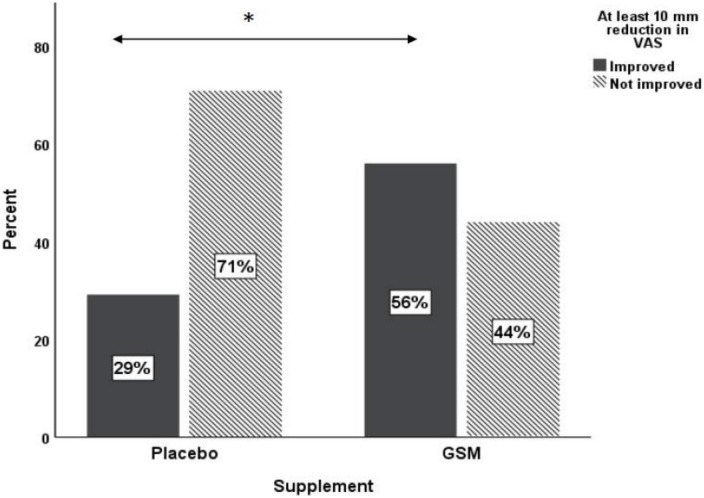
Proportion of responders [those who had ≥10 mm reduction in baseline visual analogue scale (VAS)] versus non-responders in placebo and GSM supplement groups. *Pearson’s chi-square test *P* = 0.05.

The pattern of change in VAS pain score over the study period is presented in Figure 4. There was a significant change in VAS pain score between the groups. Both unadjusted and adjusted analysis of the VAS pain score showed a greater reduction from baseline in the GSM group compared with placebo (−13.2 ± 20.3 vs. −2.9 ± 15.9, *P* = 0.03 unadjusted, and *P* = 0.04 adjusted for covariates). A significant time effect (the difference between baseline and endpoint) was found for the VAS pain score (*P* = 0.002 unadjusted). The rate of positive response or minimal clinical improvement in VAS pain score (at least 10 mm reduction) was 56% in GSM group as compared with 29% in placebo group (*P* = 0.05), as shown in Figure 5.

Further assessments of the KOOS domains focused on subjects with symptomatic knees (those with a score ≤ 86). The mean ± SD of baseline, endpoint, change in KOOS domains score over time, and difference in change between the treatment groups are presented in Table 2. The baseline level of KOOS for any of domains was not different between the groups. There was no significant change between the two groups for any KOOS domains. However, a significant time effect was found for the KOOS pain domain (*P* = 0.002 unadjusted); however, it lost its significance after adjustment for covariates (*P* = 0.3 adjusted). Both unadjusted and adjusted analysis of KOOS symptoms domain revealed a significant time effect (*P* = 0.02 unadjusted, and *P* = 0.03 adjusted) with greater improvement from baseline in the GSM group, although this change was not statistically significant between the groups (*P* = 0.6).

**TABLE 2:** Mean ± SD of knee injury and osteoarthritis outcome score (KOOS) domain scores over the 12 weeks of study across treatment groups^1^.

KOOS domains	Placebo (*n* = 18)	GSM (*n* = 21)		*P*-value*	
			
			Time effect	Treatment effect	Interaction effect
**Pain**					
Baseline	73.0 ± 14.2	79.5 ± 16.1	0.3	0.1	0.8
Endpoint	79.0 ± 16.1	86.4 ± 12.7			
Change	6.5 ± 14.6	7.4 ± 11.7			
Difference in change		0.912 (−4.4, 6.2)			
**Symptoms**					
Baseline	67.4 ± 14.7	74.0 ± 15.8	**0.03**	0.09	0.6
Endpoint	71.4 ± 17.8	81.2 ± 17.9			
Change	4.0 ± 18.7	7.2 ± 11.6			
Difference in change		3.2 (−2.0, 8.4)			
**Activities of daily living**					
Baseline	77.2 ± 13.6	81.8 ± 17.2	0.2	0.3	0.8
Endpoint	84.5 ± 15.0	88.6 ± 15.1			
Change	7.2 ± 13.1	6.8 ± 14.8			
Difference in change		−0.33 (−7.0, 6.3)			
**Sport/recreation**					
Baseline	59.8 ± 27.8	70.3 ± 26.5			
Endpoint	67.8 ± 24.7	69.0 ± 32.5	0.7	0.3	0.1
Change	8.0 ± 25.6	−1.2 ± 31.0			
Difference in change		−9.2 (−23.4, 4.8)			
**Quality of life**					
Baseline	49.8 ± 15.2	59.8 ± 22.7			
Endpoint	61.9 ± 22.3	70.3 ± 22.2	0.1	0.1	0.8
Change	12.1 ± 22.1	10.4 ± 18.3			
Difference in change		−1.6 (−9.9, 6.7)			

Standardized scores for each of KOOS domain ranged from 0 to 100, with higher scores representing lower pain levels and a better KOOS response.

^1^Participants with cut-off scores of 86 or below were included in analysis. Mean (95% confidence interval) for difference in change between the groups (GSM vs. placebo). *The two-way repeated measure ANOVA analyses were adjusted for baseline level, compliance, paracetamol use, and season of enrolment. Significance level (*P* < 0.05) is indicated in bold.

### Evaluation of treatment on plasma cytokines

The plasma cytokines measured were present at low concentrations (pg/mL) and in some of the samples were not detectable, which were not replaced with the limit of quantification (LOQ) in order to avoid overestimating the cytokine level. The levels of cytokines showed high variability and distribution was skewed. The medians (25th and 75th percentile) of plasma cytokines at baseline and end of the study and mean ± SD of % change from baseline is presented in Table 3. There were no significant differences between the groups at each time point (baseline and endpoint) or over the time within the groups for any of the cytokines assessed by Mann-Whitney *U* test and paired *t*-test, respectively.

**TABLE 3:** Median (25th, 75th percentile) and mean ± SD of plasma cytokines at baseline, end of the study and % change from baseline and number of participants with measurement above the detection limit in each group.

Cytokines (pg/mL)	Placebo		GSM		*P*-value*
TNF-α		*N* = 20		*N* = 20	
Baseline	26.8 (16.0, 98.2)		30.6 (9.0, 66.0)		0.4
Endpoint	37.0 (8.7, 104.3)		26.4 (14.9, 51.8)		0.5
% Change	32.6 ± 158		40.3 ± 131		0.8
IL-1β		*N* = 20		*N* = 17	
Baseline	15.3 (7.0, 49.6)		9.0 (4.1, 24.2)		0.3
Endpoint	8.5 (6.4, 92)		9.3 (4.9, 14.4)		0.3
% Change	70.7 ± 268		46.6 ± 162.2		0.7
IL-6		*N* = 22		*N* = 25	
Baseline	7.0 (3.9, 15.8)		4.6 (2.3, 10.8)		0.2
Endpoint	7.9 (4.2, 18.7)		5.1 (3.4, 9.7)		0.1
% Change	59.5 ± 183		45.8 ± 129.9		0.7
IL-15		*N* = 19		*N* = 18	
Baseline	344.9 (298.8, 706.5)		336.5 (241.4, 443.3)		0.2
Endpoint	376.3 (274.3, 822.7)		382.8 (288.6, 425.2)		0.5
% Change	7.7 ± 289.8		18.9 ± 231.6		0.3
IL-18		*N* = 22		*N* = 23	
Baseline	152.4 (88.7, 168.7)		114.8 (68.6, 199.9)		0.6
Endpoint	116.4 (104.2, 185.6)		105.0 (78.6, 159.7)		0.2
% Change	10.0 ± 54.4		6.6 ± 52.1		0.8
IL-4		*N* = 21		*N* = 24	
Baseline	47.6 (29.9, 86.0)		45.8 (20.2, 83.9)		0.4
Endpoint	41.4 (23.2, 149.7)		46.7 (20.2, 97.0)		0.4
% Change	49.2 ± 167.2		38.5 ± 137.0		0.8
IL-10		*N* = 20		*N* = 19	
Baseline	4.7 (2.5, 12.2)		3.4 (2.1, 7.3)		0.4
Endpoint	4.9 (2.3, 13.5)		4.3 (1.9, 6.3)		0.4
% Change	23.9 ± 91.2		17.4 ± 81.9		0.8

*No significant difference between GSM vs. placebo at baseline and endpoint using Mann-Whitney *U* test. No significant difference was observed overtime (baseline vs. endpoint) within the group using paired *t*-test.

### Correlation between the cartilage degradation markers, plasma 25(OH)D, and self-reported knee injury and osteoarthritis outcome score outcomes

As shown in Table 4, no correlation could be found between serum COMP and urinary CTX-II levels (*r* = 0.103, *P* = 0.4). However, a significant negative correlation was found between urinary CTX-II levels and KOOS pain score (*r* = −0.292, *P* = 0.02), symptoms score (*r* = −0.276, *P* = 0.01), and ADL score (*r* = −0.443, *P* = 0.002). Serum COMP did not show a correlation with any of the KOOS domain scores.

**TABLE 4:** Correlation between urinary C-telopeptide of type II collagen (CTX-II), serum cartilage oligomeric matrix protein (COMP), and plasma 25(OH)D with visual analogue scale (VAS) pain and knee injury and osteoarthritis outcome score (KOOS) domain scores at baseline (*n* = 49).

	Urine CTX-II	Serum COMP	Plasma 25(OH)D
VAS pain score	0.132	0.01	0.150
**KOOS**			
Pain	−0.292*	−0.053	0.240
Symptoms	−0.276*	−0.006	0.201
ADL	−0.390*	−0.04	0.253
Sport/recreation	−0.222	−0.240	0.136
Quality of life	−0.318*	0.078	0.252
Urine CTX-II	–	0.103	−0.236
Serum COMP	0.103	–	0.101
Plasma 25(OH)D	−0.236	0.101	–

Values represent Pearson correlation coefficients.

*Indicates significance at *P* ≤ 0.05.

Correlations between baseline plasma 25(OH)D and cartilage markers and KOOS domain scores were also evaluated. Plasma 25(OH)D levels did not show any significant correlation with urinary CTX-II or serum COMP levels. However, a weak and insignificant positive correlation appeared between plasma 25(OH)D and KOOS pain (*r* = 0.240, *P* = 0.09), ADL score (*r* = 0.253, *P* = 0.07) and quality of life (*r* = 0.252, *P* = 0.08).

As expected, the baseline plasma CTX-I level were shown to be negatively correlated with whole body mass density (*r* = −0.355 *P* = 0.01) and not with any of cartilage markers.

### Medication and analgesic use over the study period

Participants continued their current medications prescribed to them by their physician for management of chronic diseases throughout the trial. The type of medications included cholesterol-lowering agents, anti-hypertensive medications, proton pump inhibitors, anti-depressants, and thyroid medications. The majority of subjects received COVID-19 vaccinations during the study. In the GSM group, 36% (*n* = 9) of subjects used analgesic medication (Paracetamol) for joint symptoms, compared with 20.8% (*n* = 5) in the placebo group. During the study, 8% (*n* = 2) of subjects in the GSM and 16.6% (*n* = 4) in the placebo group took NSAIDs (diclofenac sodium and ibuprofen) for headaches or migraine.

### Safety and adverse events

Baseline blood analyses indicated that total cholesterol and LDL were above the normal range and elevated in both groups. HbA1c was also close to the upper cut-off of the normal range. Cholesterol and blood glucose may be elevated with obesity and menopause. There was no difference between the groups at the end of the study for the lipid profile other than HDL, liver enzymes and kidney function tests (Supplementary Table 3). Of all 49 subjects who completed the study, 20% (*n* = 5) of participants in the GSM group and 8.3% (*n* = 2) in the placebo group reported adverse events that occurred on a few occasions during the intervention. The most frequent adverse event reported was mild to moderate indigestion and reflux (GSM, *n* = 3 and placebo, *n* = 1) for which two participants took omeprazole. Other adverse events include mild abdominal pain (GSM, *n* = 1 and placebo *n* = 1), and nausea (GSM, *n* = 1).

### Compliance and adherence to study supplement

A generally high adherence was observed (98%) in both groups. Two participants (one in GSM, and one in placebo) were not able to complete the final visit on week 12 due to COVID-19 restrictions and their final visit was postponed to week 16. Compliance was also confirmed by the analysis of *n*-3 PUFA concentration in plasma and RBC membranes. The mean plasma and RBC concentrations of EPA, DHA and total *n*-3 L-C PUFA at baseline, end of the study and change from baseline are presented at Table 5. Plasma EPA and total *n*-3 L-C PUFA (g/L) increased by 0.57 ± 1.4 and 0.32 ± 5.0 g/L, respectively, in the GSM group while they decreased in the placebo group by −0.31 ± 1.2 and −2.07 ± 5.1 g/L (*P* ≤ 0.05). The plasma DHA concentration decreased in both groups although the decrease was greater in the placebo group compared to GSM (−1.6 ± 2.3 vs. −0.27 ± 1.8 g/L, *P* = 0.03). Regarding the *n*-3 PUFA in RBC, at the end of the study a higher level of DHA was shown in the GSM group compared to placebo (0.81 ± 0.38 vs. 0.62 ± 0.32 g/L, *P* = 0.07). In addition, RBC omega-3 index tended to increase in the GSM group while reduced in placebo (0.13 ± 1.5 vs. −0.02 ± 2.8). However, there was no significant change between the groups or over the study period. Overall, the measurement of *n*-3 PUFA in plasma and RBC indicated a good compliance rate and confirmed the capsule count.

**TABLE 5:** Mean ± SD in plasma and red blood cell (RBC) of eicosapentaenoic acid (EPA), docosahexaenoic acid (DHA) and total *n*-3 long chain-polyunsaturated fatty acids (*n*-3 LC-PUFA) at baseline, end of study and change from baseline.

Plasma fatty acid (g/L)	Placebo (*n* = 24)	GSM (*n* = 25)	*P*-value*
**Eicosapentaenoic acid (EPA)**			
Baseline	3.1 ± 1.1	3.2 ± 1.0	0.9
Endpoint	2.8 ± 0.9	3.8 ± 1.3	**0.008**
Change	−0.31 ± 1.2	0.57 ± 1.4	**0.02**
**Docosahexaenoic acid (DHA)**			
Baseline	7.2 ± 2.9	6.5 ± 2.0	0.3
Endpoint	5.6 ± 1.6	6.2 ± 2.0	0.2
Change	−1.6 ± 2.3	−0.27 ± 1.8	**0.03**
**Total *n*-3 LC- PUFA**			
Baseline	16.4+5.9	15.1 ± 4.3	0.3
Endpoint	13.7 ± 3.9	15.4 ± 4.3	0.1
Change	−2.07 ± 5.1	0.32 ± 5.0	**0.03**

**RBC fatty acid (g/L)**	**Placebo (*n* = 24)**	**GSM (*n* = 23)**	*P*-value*

**Eicosapentaenoic acid (EPA)**			
Baseline	0.39 ± 0.3	0.51 ± 0.34	0.2
Endpoint	0.62 ± 0.32	0.81 ± 0.38	0.07
Change	0.32 ± 0.38	0.30 ± 0.42	0.5
**Docosahexaenoic acid (DHA)**			
Baseline	1.9 ± 1.1	2.31 ± 2.2	0.3
Endpoint	2.7 ± 1.1	3.1 ± 1.6	0.3
Change	0.7 ± 1.4	0.8 ± 1.8	0.8
**Total *n*-3 LC- PUFA**			
Baseline	3.7 ± 2.3	4.5 ± 2.2	0.2
Endpoint	5.2 ± 2.0	6.1 ± 2.9	0.2
Change	1.4 ± 3.0	1.6 ± 3.3	0.8
**Omega-3 index (%)**			
Baseline	5.17 ± 2.5	5.8 ± 1.7	0.2
Endpoint	5.14 ± 2.0	5.9 ± 1.9	0.1
Change	−0.02 ± 2.8	0.13 ± 1.5	0.8

Values are reported as mean ± SD. The total *n*-3 PUFA including alpha-linolenic acid (ALA, 18:3 *n*-3), stearidonic acid (SDA, 18:4 *n*-3), eicosatetraenoic acid (ETA 20:4 *n*-3), EPA, docosapentaenoic acid (DPA,22:5 *n*-3), and DHA. Omega-3 index is content of EPA + DHA in RBC membranes expressed as a percent of total fatty acids. *The difference between group at baseline, endpoint and change from baseline were determined by Student’s *t*-test. *P* ≤ 0.05 is indicated in bold.

## Discussion

This study was the first to evaluate the effect of whole meat GSM powder on cartilage metabolism in overweight/obese postmenopausal women with joint pain and discomfort using the biomarkers of type II collagen degradation (CTX-II) and non-collagen cartilage degradation (COMP). The present study revealed the change in the urinary CTX-II/Cr was not significant between the two treatment groups. The result showed it was moderately decreased following 12 weeks of GSM treatment but notably elevated in the placebo group. This effect was observed in subjects with symptomatic knees, as urinary CTX-II levels were significantly different between the treatment groups at week 6 and end of study. This study also showed benefits of GSM supplement over placebo for secondary outcomes of VAS pain. The improvement for VAS pain in GSM group was 13 mm which is considered as clinically meaningful. However, GSM supplementation did not influence the level of circulating inflammatory cytokines.

The lack of effect of GSM on urinary CTX-II could be due to high levels of urinary CTX-II at baseline (571.6 ± 415.6 ng/mmol Cr), which were higher than the values from a previous study (511.92 ± 486.21 ng/mmol Cr) using the same ELISA kit in elderly females with knee OA (age 64.45 ± 10.6 years) (38). Thus, due to high levels of urinary CTX-II, a notable reduction may not have been detected after 12 weeks of GSM treatment, and longer duration may result in a more significant effect. Of note, high levels of urinary CTX-II could be due to its high variability as a recent meta-analysis reported the mean levels were between 129 and 345 ng/mmol Cr in healthy adults (39). A significant difference in levels of urinary CTX-II between groups at follow-up and end of trial in participants with symptomatic knees was found, which may suggest these groups within the population obtain a larger cartilage-protective effect by GSM assessed through reduction of type II collagen degradation. However, the reason for elevation in level of urinary CTX-II observed in the placebo group during the intervention is not clear. This might be due to a withdrawal effect of chondroprotective supplements and dietary restrictions for omega-3 rich foods during the study by these participants. It worth mentioning that urine samples were collected following overnight fasting, although it is possible that it does not reflect the acute chondroprotective effect of GSM. However, it can be proposed that the chondroprotective effect of GSM is not acute when consumed over a long period of time and could be reflected in general body fluids.

The effect of GSM on urinary CTX-II is consistent with a previous rat study which revealed a lower concentrations of serum CTX-II in diet-induced obese rats fed with GSM powder (22). The concentration of serum CTX-II is in line with urine CTX-II in rats (40); however, the serum CTX-II assay it is not the same as urine level in humans. Although the serum level has less analytical and biological variation than urine, we applied urine CTX-II in this study because it is known to have better clinical relevance than serum. Urine CTX-II has been used to discriminate OA patients from non-OA, and is strongly associated with clinical variables such as Kellgren-Lawrence grading (KLG) grade and knee OA symptoms (41).

In contrast, GSM supplementation did not affect the levels of serum COMP, a degradation marker from non-collagen components of cartilage. This lack of effect could be partly explained by the insignificant correlation between urinary CTX-II and serum COMP, suggesting that these markers are unlikely to change in parallel within the body after treatment. The levels of both urine CTX-II and serum COMP have shown a significant increase post-menopause; however, the increase tended to be less apparent for serum COMP and its level was generally lower in women than men within a similar age range (42).

This study showed a clinically significant reduction of pain on VAS (over 10 mm reduction) in favor of GSM. To our knowledge there are only two recently published clinical trials of whole GSM powder [16, 37]; both showed improvement in pain and knee OA symptoms measured by VAS pain and Western Ontario and McMaster Universities Osteoarthritis Index (WOMAC). These studies used the freeze-dried whole GSM powder product GlycOmega™ PLUS, which was administrated at the same dose (3 g/day) and duration as our study. The first trial was a single arm with duration of 8 weeks resulting in a significant improvement in WOMAC total and sub-scores (pain, stiffness, and physical function) in knee OA patients (43). In the second trial, GSM powder was compared to glucosamine sulfate in a 12 week intervention where both supplements showed equal effectiveness on the aforementioned outcome measures (18). Our recent systematic review on the existing clinical trials concluded that both GSM lipid extract or whole meat powder products provide a clinically meaningful improvement in VAS pain for OA symptoms (44). In terms of knee related symptoms, the GSM group showed a greater improvement in KOOS symptoms domain overtime, although the change was not significant and did not reach the suggested minimal clinical improvement (at least eight points improvement) (32).

In our study, the urinary CTX-II levels showed an inverse correlation with some of the KOOS domains scores, showing that a higher level of knee-related problems was reflected in a higher level of urinary CTX-II. This is in accordance with previous studies using the patient-reported outcome of WOMAC index (38). This observed correlation may explain the beneficial effect of GSM on both urinary CTX-II and the KOOS domains, while COMP levels were not affected.

The level of plasma CTX-I at baseline was comparable between the two groups and within the range reported by a previous study (0.45 ± 0.1 μg/L) (45).

The main compounds with bioactive properties in GSM are lipids PUFA, EPA, and DHA that are known for their anti-inflammatory effect by inhibiting the COX enzyme, which most likely explain the analgesic and pain-reducing effect of GSM (19). Matrix metalloproteinases (MMPs), particularly MMP-13, are primary enzymes involved in the degradation of type II collagen and inhibition of MMP-13 has been a target in OA treatment (46). There is *in vitro* evidence showing that omega-3 from GSM oil extract down-regulates the expression of catabolic genes MMP-1, MMP-3, and MMP-13, while up-regulating the expression of anabolic genes that encode aggrecan and collagen type II-alpha (AGG and COL2A1) (47). It must be noted that whole meat GSM powder also contains cartilage protective and glycosaminoglycan such as glucosamine and chondroitin (3% of whole GSM powder extract) (48). These compounds been shown to have inhibitory effects on MMP production *in vitro* (49). Three months supplementation with glucosamine (1.5 and 3 g/day) has been shown to reduce the urinary levels of CTX-II in athletes (50). Although the glycosaminoglycan content in the dose provided in this study was ∼90 mg/day which is less than the effective dose reported by the previously mentioned study (50), whole GSM powder is a blend of omega-3 PUFA, glucosamine and chondroitin and several other bioactive components; therefore, it can be speculated that GSM powder can provide additive chondroprotective effects through regulation of MMPs which result in suppression of type II collagen degradation. Further *in vitro* studies are required to elucidate detailed molecular mechanisms.

Greenshell mussel supplementation did not significantly affect the inflammatory or anti-inflammatory cytokines as compared with placebo. Similarly, no significant change in circulating cytokine levels were observed in the previous study of obese rats fed with a GSM-enriched diet (22). Changes in circulating markers of inflammation such as TNF-α, IL-6, CRP, and adhesion molecules have not been observed in previous studies among healthy elders (51), or healthy obese postmenopausal women supplemented with omega-3 PUFA or fish oil supplements (52).

One obvious strength of the current study is its novelty, assessing the effect of GSM supplementation on cartilage degradation markers in human subjects for the first time. Secondly, this study assessed EPA, DHA, and total omega-3 PUFA in plasma and RBC to confirm compliance of the study participants.

Some limitations of our study must be acknowledged. Firstly, participants were not screened by radiographic evidence to detect OA due to resource limitation. Observing the high level of urinary CTX-II raised the possibility of participants having established OA which would not be unexpected as most participants were older women with moderate to severe pain. Stratifying based on age and adjusting the baseline VAS pain score helped to negate this effect to some extent. Secondly, higher levels of urinary CTX-II in women than men were reported in a previous study (38), which was partly explained by the effects of menopause, and thus a male population may respond better to GSM supplementation. The effects of diurnal variation on biomarker levels should be taken into consideration. The urinary CTX-II has the highest level in the morning which decreases 4 h after arising from bed and then remains stable till after 12 h (53). The timing of urine sample collection in our study was in the morning between 8 and 9 a.m. which was most convenient for participants but is considered the highest phase of diurnal variation. Moreover, the urine sample was collected according to standard methods for CTX-II assessment, although a 24-h urine sample collection was proposed to fully monitor the chondroprotective effect of active agents (54). However, in this study 24-h samples were not collected due to the potential burden on participants.

No minimum level of pain was set as inclusion criteria, and thus half of the study participants (53%) had mild symptoms (below 30 mm) at baseline. The study subjects were predominantly of New Zealand-European ethnicity. Previous research has reported significant ethnicity-based differences in the experience of pain and treatment response among OA patients (55). Whether our findings are applicable to individuals with only severe symptoms or from other ethnicities is unclear and requires further research. This study used KOOS questionnaire for outcome measure which is specifically for the knee joint because the knee OA is dominant among postmenopausal women (56), and majority of this study participants were experiencing knee pain. This study was not limited to individuals with only knee pain in order to generalize the findings to individuals with pain at other joint sites. This also allowed to have the study population that represent the population of postmenopausal women with affected joints at different site. Finally, this study focused on a limited number of cartilage degradation markers and lacked assessment of a cartilage synthesis marker. It was originally proposed to determine the level of C-terminal propeptide type II collagen (C-propeptide, also referred as CPII), a commonly measured collagen type II synthesis biomarker (57). However, delivery of the assay kits was severely delayed due to COVID-19 impacts on global transport systems, and the kits when received, proved to be unusable. We acknowledge that the current results identifying effects of GSM supplement on urine CTX-II should be interpreted cautiously and need to be evaluated against other cartilage markers, specifically the ratio of CTX-II/CPII. However, these limitations do not negate our overall conclusions.

## Conclusion

In summary, the present study revealed that whole meat GSM powder did not change the cartilage metabolism evaluated by urinary CTX-II and serum COMP levels in overweight/obese postmenopausal women, however, in those with knee symptoms, urinary CTX-II was decreased. GSM supplementation was effective in clinically improving joint pain; however, it did not impact knee-related symptoms and the level of inflammatory cytokines.

## Data availability statement

The raw data supporting the conclusions of this article will be made available by the authors, without undue reservation.

## Ethics statement

Massey University Human Ethics Committee approved this study: Southern A, Application 20/03. The patients/participants provided their written informed consent to participate in this study.

## Author contributions

MK, JC, and FW: conceptualization, methodology, and supervision. MA: chief investigator and writing—original draft preparation. JC, FW, and PH: writing, reviewing, and editing the manuscript. MM and HT: funding acquisition, reviewing, and editing the manuscript. All authors read and approved the final manuscript.

## Abbreviations

GSM: greenshell mussel
CTX-II: C-telopeptide of type II collagen
COMP: cartilage oligomeric matrix protein
VAS: visual analogue scale
KOOS: knee injury and osteoarthritis outcome score
OA: osteoarthritis
ER: estrogen receptors
BMI: body mass index
TNF-α: tumor necrosis factor-α
IL-1β: interleukin-1 beta
IL-6: interleukin-6
MMPs: matrix metalloproteinases
HFHS: high-fat/high-sugar
OVX: ovariectomized
RA: rheumatoid arthritis
NZPAQ-SF: New Zealand Physical Activity Questionnaire – Short Form
IPAQ: International Physical Activity Questionnaire
CV: co-efficient of variation
Cr: creatinine
CTX-1: C-terminal telopeptide of type I collagen
EPA: eicosapentaenoic acid
DHA: docosahexaenoic acid
ALA: alpha-linolenic acid
SPMs: specialized pro-resolving mediators
SDA: stearidonic acid
ETA: eicosatetraenoic acid
KLG: Kellgren-Lawrence grading
WOMAC: Western Ontario and McMaster Universities Osteoarthritis Index.
