# Supplementary material for: Effects of Greenshell™ mussel intervention on biomarkers of cartilage metabolism, inflammatory markers and joint symptoms in overweight/obese postmenopausal women: A randomized, double-blind, and placebo-controlled trial
Source: Front Med (Lausanne). 2022 Dec 5;9:1063336. doi: 10.3389/fmed.2022.1063336 (PMC9760926; doi:10.3389/fmed.2022.1063336)
Supplement: Supplementary file 1 [file Data_Sheet_1.docx]

(A)


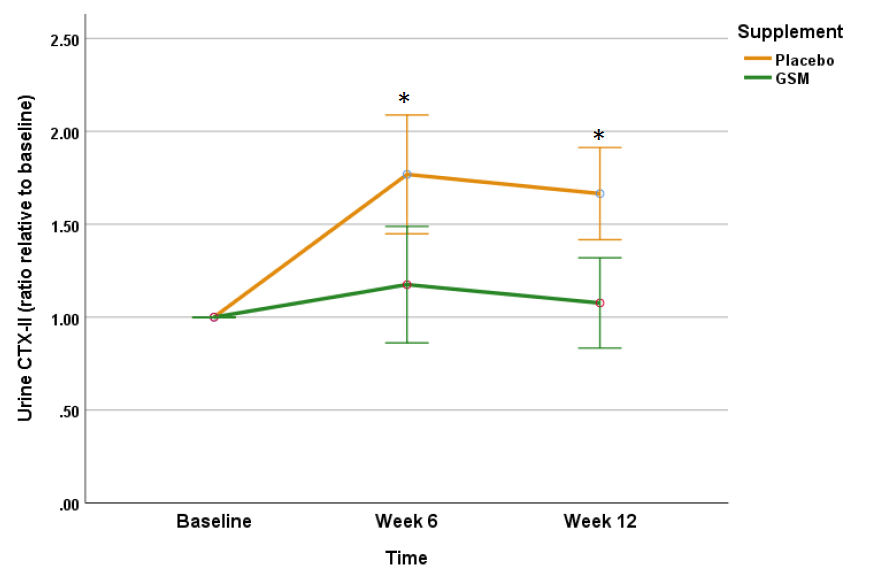


(B)


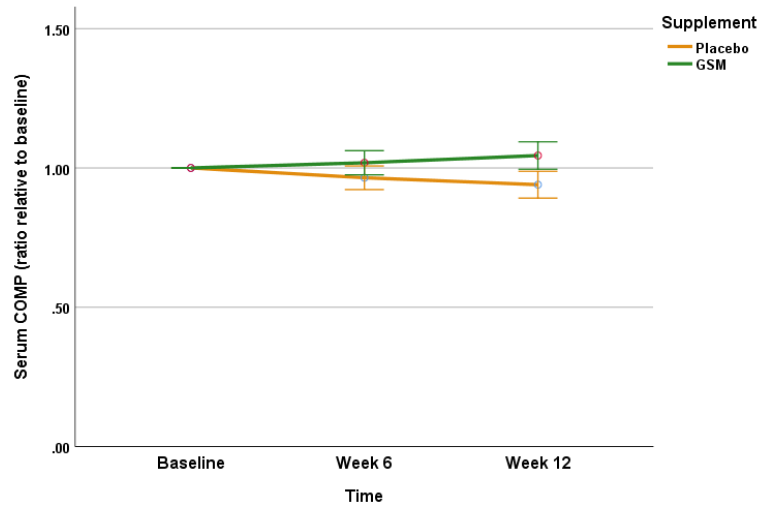


Figure S1. Patterns of change in urinary CTX-II (A) and serum COMP level (B) over the study period (baseline, follow-up, and endpoint) within each of the treatment groups. The levels are expressed as ratio relative to baseline. A significant time effect (P=0.03) for urine CTX-II (A). * Post-hoc comparison using the Tukey test showed difference between the baseline and week 6 and week 12. Placebo= orange, GSM= green. Data are expressed as mean ± standard error.

| Table S1. The proximate nutritional composition and fatty acids profile of whole greenshell mussel (GSM) powder and placebo (sunflower seed protein). | | | |
| --- | --- | --- | --- |
|  | GSM powder (g/100 g) | | Placebo (g/100 g) |
| Crude protein | | 41.4 | 24.3 |
| Carbohydrate | | 30.8 | 66.6 |
| Ash (minerals) | | 10.7 | 2.7 |
| Fat | | 10.1 | 3 |
| Moisture | | 7 | 3.4 |
| Fatty acid profile (% total fatty acids) | |  |  |
| C14:0 myristic acid | | 6.4 | 0 |
| C16:0 palmitic acid | | 16.3 | 10 |
| C16:1 palmitoleic acid | | 12.3 | 0 |
| C18:0 stearic acid | | 4.3 | 5.8 |
| C18:1n7 vaccenic acid | | 3.6 | 0.62 |
| C18:1n9c oleic acid | | 1.3 | 34.4 |
| C18:2n6c linoleic acid  C20:4n6 arachidonic acid (AA) | | 1.7  1.3 | 33.1  0 |
| C20:5n3 eicosapentaenoic acid (EPA) | | 20.7 | 0 |
| C22:5n3 docosapentaenoic acid (DPA) | | 1.1 | 0 |
| C22:6n3 docosahexaenoic acid (DHA) | | 8 | 0 |
| C20:4n3 eicosatetraenoic acid (ETA) | | 0.35 | 0 |

| Table S2. The daily energy and nutrients intake from diet (without supplement) of participants across treatment groups | | | | |
| --- | --- | --- | --- | --- |
| Daily energy and nutrients intake | | Placebo (n=24) | GSM (n= 21) |  |
| Energy (kJ) | 9339.1±3322.1 | | 9030±4469.3 |  |
| Protein (g) | 99.9±52.3 | | 93.04±31.2 |  |
| Carbohydrate (g) | 203.5±91.5 | | 193.6±94.6 |  |
| Fat (g) | 101.1±44.5 | | 100.9±68.8 |  |
| SFA (g) | 38.9±18 | | 40.2±30.2 |  |
| MUFA (g) | 35.6±22 | | 35.4±22.8 |  |
| PUFA (g) | 14.7±7.2 | | 14.8±15.2 |  |
| EPA (g) | 0.04±0.05 | | 0.03±0.04 |  |
| DHA (g) | 0.04±0.06 | | 0.03+0.08 |  |
| Calcium (mg) | 1442.3±1797.1 | | 1061.0±509.2 |  |
| Sodium (mg) | 2413.7±758 | | 2282.0±910.4 |  |
| Potassium (mg) | 7524.0±14524 | | 5357.4±1178.8 |  |
| Iron (mg) | 16.2±17.8 | | 11.2±5.0 |  |
| Zinc (mg) | 13.8±9.5 | | 11.5±5.9 |  |
| Magnesium (mg) | 814.0±1636.1 | | 732.2±171.9 |  |
| Dietary fibre (g) | 28.6±13.0 | | 24.7±8.2 |  |
| SFA: saturated fatty acids, MUFA: monounsaturated fatty acids, PUFA: polyunsaturated fatty acids, EPA: eicosapentaenoic acid, DHA: docosahexaenoic acid. | | | | |

| Table S3. Laboratory blood parameters for safety assessment among participants (n=47) | | | | |
| --- | --- | --- | --- | --- |
|  | Reference range ꝉ | Placebo (n=23) | GSM (n=24) | P-value ^*^ |
| Total Cholesterol (mmol/L) | 0-5.0 |  |  |  |
| Baseline |  | 6.0±0.9 | 5.6±1.0 | 0.1 |
| Endpoint |  | 6.2±0.9 | 5.9±1.0 | 0.2 |
| TG (mmol/L) | 0-2.0 |  |  |  |
| Baseline |  | 1.6±0.7 | 1.7±0.7 | 0.6 |
| Endpoint |  | 1.7±0.8 | 1.9±0.8 | 0.5 |
| LDL (mmol/L) | < 2.5 |  |  |  |
| Baseline |  | 3.6±0.8 | 3.3±1.0 | 0.2 |
| Endpoint |  | 3.6±0.8 | 3.4±1.0 | 0.4 |
| HDL (mmol/L) | 1.0-0.9 |  |  |  |
| Baseline |  | 1.6±0.4 | 1.5±0.3 | 0.1 |
| Endpoint |  | 1.7±0.4 | 1.5±0.3 | 0.05 |
| Total cholesterol/LDL ratio | < 4.5 |  |  |  |
| Baseline |  | 3.7±0.9 | 3.9±1.1 | 0.5 |
| Endpoint |  | 3.7±0.8 | 3.9±1.1 | 0.4 |
| HbA1c (mmol/mol) | 20-40 |  |  |  |
| Baseline |  | 36.9±4.1 | 37.1±2.8 | 0.8 |
| Endpoint |  | 36.8±3.9 | 37.0±3.4 | 0.8 |
| Bilirubin (µmol/L) | 2-24 |  |  |  |
| Baseline |  | 8.7±5.1 | 6.9±2.7 | 0.1 |
| Endpoint |  | 8.4±4.9 | 6.4±3.1 | 0.09 |
| ALP (U/L) | 20-110 |  |  |  |
| Baseline |  | 83.8±22.3 | 85.8±18.4 | 0.7 |
| Endpoint |  | 85.1±21 | 89.9±21.4 | 0.4 |
| GGT (U/L) | 10-35 |  |  |  |
| Baseline |  | 21.5±10.3 | 21.2±16.2 | 0.9 |
| Endpoint |  | 21.8±8.6 | 17.7±7.0 | 0.08 |
| ALT (U/L) | 0-45 |  |  |  |
| Baseline |  | 18.4±7.4 | 19.8±8.2 | 0.5 |
| Endpoint |  | 18.7±5.0 | 18.5±7.4 | 0.9 |
| AST (U/L) | 10-45 |  |  |  |
| Baseline |  | 19.4±3.2 | 22.0±6.1 | 0.08 |
| Endpoint |  | 20.5±3.8 | 21.2±6.0 | 0.6 |
| Total protein (g/L) | 65-80 |  |  |  |
| Baseline |  | 70.0±4.3 | 69.8±2.9 | 0.8 |
| Endpoint |  | 70.1±4.0 | 70.5±4.2 | 0.7 |
| Albumin (g/L) | 32-48 |  |  |  |
| Baseline |  | 38.5±2.0 | 39.2±2.3 | 0.2 |
| Endpoint |  | 38.1±2.4 | 39.0±2.0 | 0.1 |
| Calcium (mmol/L) | 2.10-2.55 |  |  |  |
| Baseline |  | 2.3±0.09 | 2.3±0.06 | 0.8 |
| Endpoint |  | 2.4±0.15 | 2.3±0.08 | 0.4 |
| Adjusted calcium (mmol/L) | 2.10-2.55 |  |  |  |
| Baseline |  | 2.4±0.09 | 2.4±0.06 | 0.5 |
| Endpoint |  | 2.4±0.13 | 2.4±0.07 | 0.3 |
| Creatinine (µmol/L) | 45-90 |  |  |  |
| Baseline |  | 74.7±11.3 | 78.2±10.2 | 0.2 |
| Endpoint |  | 73.3±11.1 | 75.5±9.5 | 0.4 |
| eGFR (ml/min/1.73 m^2^) | 60-89 |  |  |  |
| Baseline |  | 75.8±12.1 | 70.1±11.1 | 0.1 |
| Endpoint |  | 76.3±11.3 | 72.9±11.0 | 0.2 |

Values are reported as mean ± SD

ꝉ Reference ranges provided by the Palmerston North hospital-based clinical laboratory

^*^ The differences between group at baseline and endpoint were determined by Student’s t-test. Significance at P<0.05 is indicated in bold.
